# Common germ-line polymorphism of *C1QA* and breast cancer survival

**DOI:** 10.1038/sj.bjc.6605625

**Published:** 2010-03-23

**Authors:** E M Azzato, A J X Lee, A Teschendorff, B A J Ponder, P Pharoah, C Caldas, A T Maia

**Affiliations:** 1 Strangeways Research Laboratories, Department of Oncology, University of Cambridge, Cambridge CB1 8RN, UK; 2 Genetic Epidemiology Branch, Division of Cancer Epidemiology and Genetics, National Cancer Institute, National Institutes of Health, Rockville, MD 20852, USA; 3 Cancer Research UK, Cambridge Research Institute, Robinson Way, Cambridge CB2 0RE, UK; 4 Department of Oncology, Addenbrooke's Hospital, University of Cambridge, Hills Road, Cambridge CB20XZ, UK; 5 Medical Genomics Group, UCL Cancer Institute, University College London, London, UK; 6 Cambridge Experimental Cancer Medicine Centre, Li Ka Shing Centre, Robinson Way, Cambridge CB2 0RE, UK

**Keywords:** *C1QA*, breast cancer, SNP, risk, differential allelic expression

## Abstract

**Background:**

A synonymous single nucleotide polymorphism (SNP) rs172378 (A>G, Gly−>Gly) in the complement component *C1QA* has been proposed to be associated with distant breast cancer metastasis. We previously reported overexpression of this gene to be significantly associated with better prognosis in oestrogen-receptor-negative tumours. The purpose of this study was to investigate the association of rs172378 with expression of *C1QA* and breast cancer survival.

**Methods:**

We analysed the gene expression pattern of rs172378 in normal and tumour tissue samples, and further explored its involvement in relation to mortality in 2270 women with breast cancer participating in Studies of Epidemiology and Risk factors in Cancer Heredity, a population-based case–control study.

**Results:**

We found that although rs172378 showed differential allelic expression significantly different between normal (preferentially expressing the G allele) and tumour tissue samples (preferentially expressing the A allele), there was no significant difference in survival by rs172378 genotype (per allele hazard ratio (HR) 1.02, 95% CI: 0.88–1.19, *P*=0.78 for all-cause mortality; HR 1.03, 95% CI: 0.87–1.22, *P*=0.72 for breast-cancer-specific mortality).

**Conclusion:**

Our study results show that rs172378 is linked to a *cis*-regulatory element affecting gene expression and that allelic preferential expression is altered in tumour samples, but do not support an association between genetic variation in *C1QA* and breast cancer survival.

Complement is involved in the primary defence against intravascular microorganisms and has been reported to be involved in the clearance of tumour cells (Jurianz *et al*, 1999a, 1999b; Golay *et al*, 2000; Caragine *et al*, 2002; Fishelson *et al*, 2003). Recently, we have reported an association between expression of *C1QA* and prognosis in oestrogen receptor (ER)-negative breast cancer (Teschendorff *et al*, 2006; Teschendorff and Caldas, 2008) in more than one cohort. We found that ER-negative tumours with overexpression of gene *C1QA* were associated with a better prognosis.

The *C1QA* gene, located on chromosome 1p36.12, encodes for one of the components of the C1q complex. There are seven single nucleotide polymorphisms (SNPs) catalogued for *C1QA* on the NCBI database, of which there is only one common SNP (minor allele frequency >5%) located in an exon rs172378 is a synonymous SNP characterised by a G for A substitution at position 361 (A361G). This SNP has been previously reported as being associated with breast cancer metastasis to sites linked to hematogenous spread of disease (Racila *et al*, 2006) and with drug response in follicular lymphoma (Racila *et al*, 2008). In a set of 63 patients with localised breast tumours and 38 patients with metastasis, Racila *et al* (2006) reported a decreased time to metastasis for G homozygote or heterozygote individuals compared with the common AA homozygote (hazard ratio, 95% CI: 2.4, 1.1–4.1), even after adjustment for positive lymph nodes, oestrogen and progesterone receptors status.

Racila *et al.* (2006) have also reported that rs172378 correlates with decreased complement activity, which then reduces the instance of metastasis associated with breast cancer, perhaps by resulting in an inefficient clearance of apoptotic tumour cancer cells, which consequently results in the development of a more effective antibody response against the tumour. The same group previously identified a correlation between the A allele of rs172378 with lower expression of the C1QA protein (Racila *et al*, 2003). The purpose of this study was to investigate the association of this SNP with expression of *C1QA* and breast cancer survival.

## Materials and methods

### Genotyping

Genotyping was carried out using the TaqMan platform as per the manufacturer's instructions. Primers and FAM- and VIC-labelled probes were supplied directly by Applied Biosystems (Foster City, CA, USA) as Assays-by-Design. All assays were carried out as previously described (Azzato *et al*, 2008). Deviation from Hardy–Weinberg equilibrium was assessed using a χ^2^-statistic.

### Expression analysis of *C1QA*

Analysis of the expression of *C1QA* was performed in a set of blood samples (*n*=57) and normal breast tissue (*n*=5, heterozygotes for rs172378) from healthy blood donors, as well as breast tumour tissue with normal copy number for the *C1QA* locus (*n*=17, heterozygotes for rs172378). Samples were obtained from the Addenbrooke's Hospital Tissue Bank and Blood Centre, following ethical approval. Breast tumour cases were selected on the basis of having normal copy number, as determined by oligonucleotide-based array CGH analysis (Chin *et al*, 2007). In total, 25 samples were excluded on the basis of having copy number aberrations in the C1QA region, which represented approximately 25% of the total number of samples. The demographics of the samples analysed in this study is representative of the total group of samples (Supplementary Table). DNA and total RNA were extracted from these samples and cDNA was prepared with the TaqMan Reverse Transcription Reagents kit (Applied Biosystems) using random hexamers, according to the manufacturer's instructions. DNA from all samples was genotyped by TaqMan analysis, using the genotyping assay for rs172378.

Analysis of differential allelic expression was performed by quantitative real-time allele-specific PCR, using TaqMan assay, as previously described (Maia *et al*, 2009). This is the most suitable method for testing a small to medium number of SNPs in a relatively large number of samples, with high accuracy and replication. In brief, the region surrounding the SNP was amplified from cDNA of heterozygotes and the two probes, labelled differently with FAM and VIC, have a mismatch and recognised specifically each allele. During the real-time PCR reaction two signals were detected in each well, one for each allele. The quantity of each allele was extrapolated from a standard curve generated from a dilution series of heterozygote blood DNA (50 : 50 allelic ratio). Allelic expression ratios were calculated as log_2_((*G* allele (VIC))/(*A* allele (FAM))). The genotyping TaqMan assay included primers and probes within the coding region, so it was possible to use it for the analysis of allelic expression, which was performed in heterozygous samples only, using three replicates per assay.

Total expression levels of all samples, heterozygous and homozygous for both alleles, were determined using a TaqMan Gene Expression Assay (assay ID: Hs00381122_m1). Results were normalised with the total levels of expression of actin-*β*, GAPDH and HPRT.

### Study population for association study

Cases were selected from the Studies of Epidemiology and Risk factors in Cancer Heredity (SEARCH) breast cancer study, an ongoing population study of women diagnosed with breast cancer in the region of UK included in the Eastern Cancer Registration and Information Centre (ECRIC, formerly East Anglian Cancer Registry), as previously reported (Azzato *et al*, 2008). All participants in the study provided informed consent, and the study was approved by the Eastern Multicentre Research Ethics Committee. DNA was available from 4470 cases for genotyping. The samples were split into two sets (set 1, *n*=2270 and set 2, *n*=2200) to save DNA and reduce genotyping costs. Median age at diagnosis was similar for both sets (50 and 53 years old, respectively). Median time from diagnosis to blood draw was slightly longer for set 2 (18 months) than for set 1 (9 months), but the number of deaths in each set was similar (359 in set 1 and 278 in set 2). There was no significant difference in the morphology, histopathological grade or TNM stage of the cases by set.

Follow-up information and all-cause mortality details were obtained based on a combination of a follow-up through the Office of National Statistics for death notification and an active follow-up registration and every 5-years by the ECRIC and the North Thames Cancer Registry Breast cancer-specific mortality was defined as deaths where breast cancer was listed as the cause of death in Part 1 of the death certificate (Azzato *et al*, 2008). TNM stage (1, 2, 3 or 4), which is based on tumour size (T), number of positive lymph nodes (N) and the presence of distant metastasis (M) (Sobin and Wittekind, 1997), and histopathological grade (well, moderately and poorly differentiated) were obtained through ECRIC. ER status was determined by performing immunohistochemistry on paraffin-embedded sections of breast tumour using the Novocastra clone 6F11. The Allred system was used for scoring; scores >2 were considered positive (Harvey *et al*, 1999).

### Survival analysis statistical methods

Associations with all-cause and breast-cancer-specific mortality were assessed for rs172378 genotype using Cox regression analysis, modelling the time from diagnosis to death. To account for variable time from diagnosis to recruitment, we conducted analyses allowing for left truncated data in which cases were only considered at risk after the date of study entry. This generates an unbiased estimate of the HR provided the proportional hazard assumption is correct (Azzato *et al*, 2009). Follow-up was censored at the earlier of either date last known to be alive or 10 years after diagnosis, as follow-up became less reliable after 10 years. The proportional hazards assumption was evaluated by visual inspection of log-log plots, as well as tested analytically using Schoenfeld residuals (Grambsch and Therneau, 1994).

A per-allele HR was estimated for rs172378 genotype (co-dominant, log-additive model), based on the number of rare alleles (G) carried. On the basis of previous results by Racila *et al* (2006), a G dominant HR (AG/GG), relative to the common homozygote (AA), was also estimated. Statistical significance was assessed using a trend test (1 degree of freedom). Significant associations with survival in set 1 at a nominal *P*<0.10 were genotyped in set 2. Data from both sets can then be combined (*N*=4470) to jointly analyse associations. This joint analysis approach results in increased power to detect genetic associations, despite more stringent significance levels with Bonferroni correction (Skol *et al*, 2006).

For multivariate models, ER status was modelled as a dichotomous variable and age at diagnosis was modelled as a categorical variable (<40, 40–49, 50–59 and 60+). We compared individual models for both TNM stage (1, 2, 3/4) and histopathological grade fitted as either continuous or categorical variables. As the fit of each prognostic factor's models (categorical *vs* continuous) was similar, we classified these variables based on the simplest model (continuous). ER status was found to violate the proportional hazard assumption; as such, multivariate models were adjusted by age, TNM stage and histopathological grade, and stratified by ER status. A formal test of interaction between genotype and ER status (effect beyond additive) was performed by inclusion of an SNP-prognostic term. A test of heterogeneity (1 degree of freedom) was used to assess for differences between stratified parameter estimates. Statistical tests were two sided, with an *α*-level of 0.05. All analyses were performed in Intercooled Stata, version 9.2 (StataCorp LP, College Station, TX, USA).

## Results

### Gene expression analysis of *C1QA* (rs172378)

**Figure 1:**
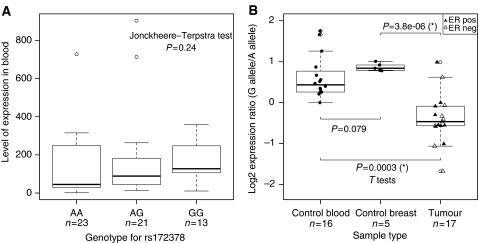
Gene expression analysis of *CI QA*. (**A**) Analysis of total level of gene expression by genotype at the rs172378 polymorphism, in blood samples from healthy control individuals. (**B**) Analysis of differential allelic expression in heterozygous samples for the rs172378 polymorphism in control blood, control breast tissue and tumour breast tissue; data points for tumour samples are shown as a closed triangle for oestrogen receptor (ER)-positive samples and as an open triangle for ER-negative samples. In both plots, the number of data points is indicated below each boxplot. Significant *t*-tests are indicated by asterisk (^*^).

The SNP rs172378 has been previously reported to have a correlation with lower levels of C1qA in serum (Racila *et al*, 2003). We analysed the correlation of total levels of *C1QA* and the rs172378 genotype in the blood of control individuals (Figure 1A). We did not find a significant trend in our sample set.

To investigate whether there were *cis*-regulatory elements affecting the expression of *C1QA*, we analysed the levels of allelic transcripts in heterozygous samples (Yan *et al*, 2002; Lo *et al*, 2003; Udler *et al*, 2007; Maia *et al*, 2009). Using allele-specific TaqMan PCR, we determined the allelic gene expression ratios (*G* allele/*A* allele) in blood and breast tissue from healthy controls heterozygous for rs172378, as well as tumour tissue from breast cancer patients. We previously reported that the *G* allele was commonly preferentially expressed in the samples from healthy individuals, but that there were no significant differences between blood and breast tissue (in blood, log_2_ mean ratio=0.61 and s.d.=0.52; in breast, log_2_ mean ratio=0.87 and s.d.=0.10; *P*=0.079) (Figure 1B), suggesting that there is a common *cis*-regulatory element for the two types of tissue (Maia *et al*, 2009). However, we now found a highly significant difference between normal breast *vs* breast tumour (*P*=3.8E−6) (Figure 1B). In fact, we found that although in the normal tissues the G allele was the preferentially expressed allele, patient tumour tissue presented preferential expression of the alternative A allele (log_2_ mean ratio=−0.39 and s.d.=0.61). We found no significant differences between the differential allelic expression ratio of tumours stratified on ER status (Figure 1B).

### Survival analysis

The characteristics of the SEARCH breast cancer cases that have been included in this report are summarised in Table 1. Cases were followed for a median of 7.75 years (from 6.72 months to 10 years). During the 13 851.3 person-years at risk there were 359 deaths before the 10 years follow-up, of which 305 were coded as due to breast cancer.

Genotyping for *C1QA* rs172378 was successful for 2168 individuals (95.2%); genotype frequencies are presented in Table 2. This SNP did not deviate from Hardy–Weinberg equilibrium in this population (*P*=0.62).

The results from the survival analyses for the co-dominant and AG/GG *vs* GG models for both all-cause and breast-cancer-specific mortality are presented in Table 3. *C1QA* rs172378 genotype did not show evidence of proportional hazard assumption violation in either model (all *P*'s>0.10). We found no evidence that overall survival varied by rs172378 genotype, either in the co-dominant model (per-rare-allele HR=1.01, 95% CI: 0.81–1.25, *P*=0.95) or the rare dominant model (HR_AG/GG *vs* AA_=0.83, 95% CI: 0.61–1.13, *P*=0.24), adjusted for age at diagnosis, TNM stage, histopathological grade and ER status. Results were consistent for breast-cancer-specific mortality. As we recently reported an association between *C1QA* expression and survival in ER-negative breast cancer, we also performed adjusted analyses stratified by ER status. No statistically significant associations were observed and there was no evidence of the HR varying by ER status (all heterogeneity *P*>0.10). In addition, formal tests of interaction by ER status were non-significant in all the models (all *P*'s>0.10). As no association reached nominal level of significance in set 1 (all *P*'s>0.10), we did not genotype this SNP in set 2.

## Discussion

The complement system and its inhibitors have been shown to be important in clearing tumour cells (Jurianz *et al*, 1999a, 1999b; Golay *et al*, 2000; Caragine *et al*, 2002; Fishelson *et al*, 2003). Recently, we showed that overexpression of *C1QA* in ER-negative basal-like breast cancer patients, which have the poorest prognosis (Sorlie *et al*, 2001), is associated with better outcome (Teschendorff *et al*, 2006, 2007; Teschendorff and Caldas, 2008).

The A allele of *C1QA* polymorphism rs172378 has been associated with decreased complement activity, resulting in reduced metastasis associated with breast cancer (Petry and Loos, 2005; Racila *et al*, 2006). The decrease in complement activity is suggested to result in less clearance of apoptotic tumour cells (Racila *et al*, 2003), and in a more effective antibody response against the tumour. Also, this same allele has been reported to associate with lower levels of serum C1qA protein (Racila *et al*, 2003). Therefore, we hypothesised that the A allele, or another allele in tight linkage disequilibrium, could have a regulatory role and could correlate with lower expression of C1qA.

We investigated the association of the rs172378 with gene expression in normal and patient samples. We found no correlation between the genotype at rs172378 and the level of expression of *C1QA* in fresh blood samples (*P*=0.07), but there was a trend towards higher mean total expression of C1qA in individuals with the G allele. Previously, when we examined the same samples for differential allelic expression we found that in both fresh blood and normal breast tissue there was consistent preferential expression of the G allele (log_2_ mean fold ratio G/A=0.87), which indicates that rs172378 is linked to a functional *cis*-regulatory element (Maia *et al*, 2009). This *cis*-element may be situated within or very close to the gene, as the linkage disequilibrium block where rs172378 lies is 11.9 kb long and includes the whole gene (3.1 kb) as well as up- and downstream regions. This finding suggests that the reported correlation between the A allele and lower levels of C1qA in serum could be associated not with translational regulation, but instead with transcriptional regulation.

Now we found that in tumour samples differential expression was also present, but the majority preferentially expressed the A allele (log_2_ mean fold ratio G/A=−0.39 for tumours), which is associated with less *C1QA* expression. The change in the preferentially expressed allele from control to tumour tissue remains unexplained. The change observed in tumour samples could be caused by the interaction of the same *cis*-element with different transcription factors when in the tumour environment, or could be a consequence of a different functional polymorphism specific to the tumour samples. This could also suggest that a less efficient complement activity could be associated with tumour development, due to a less efficient clearance of tumour cells. However, *C1QA* expressed by other tissues, for example liver, could produce a compensation effect.

Information on the ER status of the analysed tumours was available, and although the difference is not statistically significant (*t*-test *P*=0.26) there seems to exist a trend for ER-positive patients to have a higher differential allelic expression ratio, meaning a higher contribution of the G allele to the total expression of *C1QA*. We did not have sufficient ER-negative tumours to perform a correlation analysis of *C1QA* differential expression and clinical data, but all data, ours and of others, seem to suggest the lower expression of *C1QA*, which is associated with preferential expression of the A allele, could be linked to the worst prognosis associated with ER-negative tumours.

Next we evaluated the impact of rs172378 on breast cancer survival among women from the East Anglian region of the UK. This is a large-sized population-based study, characterised by a long and systematic follow-up. We have found no evidence that rs172378 is associated with outcome after a diagnosis of breast cancer, including stratification by ER status. Under our staged design, assuming a co-dominant model, a minor allele frequency of 0.44 and a joint analysis *α* level of 0.05, we had 97% power to detect an HR of 1.3. Power was not as good for the dominant (AG/GG *vs* AA) model, where we had 72% power to detect the same effect; however, power is 100% for HRs above 1.5, leaving our study more than adequately powered to detect a dominant effect similar to the one reported by Racila *et al* (2008). On the basis of our earlier finding that *C1QA* expression was associated with survival in ER-negative tumours, we stratified our sample by ER status. As ER-negative tumours are the minority of our cases (*N*=363), power to detect differential survival in this subgroup is somewhat less: assuming a co-dominant model, we have 86% power to detect an HR of 1.5 and 100% power to detect an HR of 1.7 or higher. Further, due to limited treatment information about these patients, we did not evaluate the effect of rs172378 on survival in treatment subgroups.

Overall, our data do not support the association of rs172378 with survival previously reported, but we have identified the existence of a *cis*-regulatory genetic variation that affects the expression of *C1QA*, and could explain the previously reported association of the A allele with lower C1qA protein in serum.

## Figures and Tables

**Table 1:** SEARCH participant characteristics

Total number of subjects	2270	
Total time at risk (years)	13851.3	
Median follow-up (years)^a^	7.75	(0.56–10)^b^
Median time at risk (years)	6.47	(0.10–9.64)^b^
Median time from diagnosis to study entry (years)	0.73	(0.00–8.64)^b^
Number of deaths	359	
Annual mortality rate	0.026	(0.023–0.029)^c^
5-year survival rate	0.88	(0.86–0.89)^c^
Median age at diagnosis (years)	50.2	(25–69)^b^
		
*Age at diagnosis*		
<40	212	9.3%
40–49	753	33.2%
50–59	997	43.9%
60+	308	13.6%
		
*Histopathological grade*		
Well differentiated	437	19.3%
Moderately differentiated	788	34.7%
Poorly differentiated	505	22.3%
Unknown	540	23.8%
		
*Morphological type*		
Ductal	1674	73.7%
Lobular	351	15.5%
Other	222	9.8%
Unknown	23	1.0%
		
*Clinical stage*		
1	1114	49.1%
2	987	43.5%
3 or 4	110	4.9%
Missing	59	2.6%
		
*ER Status*		
ER negative	363	16.0%
ER positive	856	37.7%
Missing	1051	46.3%

Abbreviation: ER=oestrogen receptor.

a Follow-up censored at 10 years.

b Range of variable.

c 95% CI.

**Table 2:** C1qA rs172378 genotype frequency in SEARCH study

**Genotype**	***N***	**Frequency (%)**
AA	822	36.2
AG	1031	45.4
GG	309	13.6
Missing	108	4.8
Total	2270	
MAF	0.44	

Abbreviation: MAF=minor allele frequency.

**Table 3:** Survival analysis

**Model**	**HR**	**(95% CI)**	***P***	**ER status interaction *P*** a	**ER status heterogeneity *P*** b
*All-cause mortality*					
*Co-dominant*					
All tumours (unadjusted)	1.02	(0.88–1.19)	0.78		
All tumours (adjusted)^c^	1.01	(0.81–1.25)	0.95		
ER+ tumours only^d^	1.17	(0.82–1.66)	0.39		
ER− tumours only^d^	0.94	(0.71–1.24)	0.66	0.74	0.34
					
*AG/GG* vs *AA*					
All tumours (unadjusted)	0.92	(0.74–1.15)	0.74		
All tumours (adjusted)^c^	0.83	(0.61–1.13)	0.24		
ER+ tumours only^d^	0.80	(0.50–1.28)	0.36		
ER− tumours only^d^	0.87	(0.57–1.32)	0.52	0.54	0.80
					
*Breast-cancer-specific mortality*					
*Co-dominant*					
All tumours (unadjusted)	1.03	(0.87–1.22)	0.72		
All tumours (adjusted)^c^	1.02	(0.81–1.28)	0.89		
ER+ tumours only^d^	1.07	(0.72–1.59)	0.75		
ER− tumours only^d^	1.02	(0.77–1.37)	0.87	0.72	0.87
					
*AG/GG* vs *AA*					
All tumours, unadjusted	0.92	(0.73–1.16)	0.47		
All tumours (adjusted)^c^	0.84	(0.60–1.18)	0.33		
ER+ tumours only^d^	0.75	(0.44–1.25)	0.27		
ER− tumours only^d^	0.96	(0.61–1.50)	0.84	0.31	0.48

Abbreviation: CI=confidence interval; ER=oestrogen receptor; HR=hazards ratio.

HRs, 95% CIs and *P*-values under co-dominant and dominant C1qA rs172378 genotype models for all-cause and breast-cancer-specific mortality.

a Formal test for interaction (effect beyond additive) between C1qA genotype and ER status.

b One degree of freedom *χ*^2^-test for heterogeneity between ER+/− tumor HRs.

c Models adjusted for age at diagnosis (<40, 40–49, 50–59, 60+), TNM stage, histopathological grade and stratified by ER status.

d Models adjusted for age at diagnosis (<40, 40–49, 50–59, 60+), TNM stage, histopathological grade.
