# Supplementary material for: Common germ-line polymorphism of C1QA and breast cancer survival
Source: Br J Cancer. 2010 Mar 23;102(8):1294–9. doi: 10.1038/sj.bjc.6605625 (PMC2856004; doi:10.1038/sj.bjc.6605625)
Supplement: Supplementary Table [file 6605625x1.doc]

Supplementary Table. Demographics Comparison C1QA copy-number normal vs altered

| **Total Number of Samples** | **Normal** | **Altered** | **P-Value** |
| --- | --- | --- | --- |
| ER Status |  |  | 1 |
| ER negative | 48 | 9 |  |
| ER positive | 96 | 17 |  |
| Histopathological grade |  |  | 0.68 |
| 1 | 35 | 6 |  |
| 2 | 50 | 7 |  |
| 3 | 59 | 13 |  |
| Clinical Stage |  |  | 0.43 |
| 1 | 99 | 20 |  |
| 2 | 32 | 3 |  |
| 3 | 13 | 3 |  |
| VI |  |  | 0.81 |
| 1 | 37 | 6 |  |
| 2 | 104 | 20 |  |
| NPI |  |  | 0.54 |
| Size |  |  | 0.29 |
|  |  |  |  |
| **Samples Used in this Study** |  |  |  |
| ER Status |  |  | 0.73 |
| ER negative | 4 | 8 |  |
| ER positive | 12 | 17 |  |
| Histopathological grade |  |  | 0.43 |
| 1 | 6 | 6 |  |
| 2 | 5 | 6 |  |
| 3 | 5 | 13 |  |
| Clinical Stage |  |  | 0.22 |
| 1 | 9 | 20 |  |
| 2 | 4 | 2 |  |
| 3 | 3 | 3 |  |
| VI |  |  | 0.72 |
| 1 | 5 | 6 |  |
| 2 | 11 | 19 |  |
| NPI |  |  | 0.54 |
| Size |  |  | 0.39 |

ER – Oestrogen Receptor Status

VI – Vascular Invasion

NPI – Nottingham Prognostic Index
